# Longitudinal Study of Dynamic Epidemiology of Extended-Spectrum Beta-Lactamase-Producing Escherichia coli in Pigs and Humans Living and/or Working on Pig Farms

**DOI:** 10.1128/spectrum.02947-22

**Published:** 2023-01-17

**Authors:** Wietske Dohmen, Apostolos Liakopoulos, Marc J. M. Bonten, Dik J. Mevius, Dick J. J. Heederik

**Affiliations:** a Division of Environmental Epidemiology, Institute for Risk Assessment Sciences, Utrecht University, Utrecht, The Netherlands; b Department of Bacteriology and Epidemiology, Wageningen Bioveterinary Research, Lelystad, The Netherlands; c Department of Medical Microbiology, University Medical Center Utrecht, Utrecht, The Netherlands; d Department of Infectious Diseases and Immunology, Faculty of Veterinary Medicine, Utrecht University, Utrecht, The Netherlands; University of Saskatchewan

**Keywords:** *Escherichia coli*, gene typing, *bla*_CTX-M-1_, plasmid typing, IncI1, MLST, pig farming

## Abstract

Extended-spectrum beta-lactamase (ESBL)-producing *Enterobacterales* have been increasingly isolated from pigs, highlighting their potential for transmission to humans living and/or working within pig farms. As longitudinal data on the prevalence and the molecular characteristics of such isolates from the high-risk farming population remain scarce, we performed a long-term study on 39 Dutch pig farms. Fecal samples from pigs, farmers, family members, and employees were collected during four sampling occasions with a 6-month period. The presence of ESBL-producing *Enterobacterales* and their molecular characteristics (ESBL gene, plasmid, and sequence types) were determined by standard methods. Data on personal and farm characteristics were collected using questionnaires. ESBL-producing Escherichia coli was present in pigs at least once for 18 of 39 farms and in 17 of 146 farmers, family members, and/or employees. Among these 417 E. coli isolates, *bla*_CTX-M-1_ was the most frequently observed ESBL gene in pigs (*n* = 261) and humans (*n* = 25). Despite the great variety in plasmid (sub)types and E. coli sequence types (STs), we observed genetic similarity between human- and pig-derived isolates in (i) ESBL gene, plasmid (sub)type, and ST, suggesting potential clonal transmission in seven farms, and (ii) only ESBL gene and plasmid (sub)type, highlighting the possibility of horizontal transfer in four farms. Five pig farmers carried ESBL producers repeatedly, of whom two carried an identical combination of gene, plasmid (sub)type, and ST over time. Human ESBL carriage was associated with both presence of ESBL producers in pigs and average number of hours working on the pig farm per week, while prolonged human carriage was observed only incidentally.

**IMPORTANCE** Extended-spectrum beta-lactamase (ESBL)-producing Escherichia coli represents a public health hazard due to reduced therapeutic options for the treatment of infections. Although direct contact with pigs is considered a risk factor for human ESBL-producing E. coli carriage through occupational exposure, nationwide data regarding the occurrence of such isolates among pigs and humans living and/or working on farms remain scarce. Therefore, we determined (i) the longitudinal dynamics in prevalence and molecular characteristics of ESBL-producing E. coli in Dutch pig farmers and their pigs over time and (ii) the potential transmission events between these reservoirs based on genetic relatedness and epidemiological associations in longitudinal data. Our data suggesting the possibility of clonal and horizontal dissemination of ESBL-producing Escherichia coli between pigs and pig farmers can be used to inform targeted intervention strategies to decrease the within-farm human exposure to ESBL-producing E. coli.

## INTRODUCTION

Extended-spectrum beta-lactamase (ESBL)-producing *Enterobacterales* are a burden for public health. Infections with ESBL-producing *Enterobacterales* affect morbidity, mortality, and health care costs (1, 2). Livestock, including pigs, can carry ESBL-producing *Enterobacterales* (3). Given the direct contact of humans living and/or working with pigs, transmission is likely to occur. A limited number of cross-sectional studies reported this potential transmission between pigs and pig farmers (4–9). However, the longitudinal within-farm dynamics of the prevalence and potential transmission of ESBL-producing *Enterobacterales* remains unknown. In addition, while the molecular characteristics and the duration of carriage of ESBL-producing *Enterobacterales* have been described for the general population, travelers, and health care setting (10–13), longitudinal data from other high-risk populations, such as farming communities, are still sparse.

Hence, we conducted a nationwide and longitudinal study on a large number of farms focusing on both pigs and pig farmers sampled between 2011 and 2013 in order to delineate (i) the prevalence, persistence and genetic characteristics of ESBL-producing Escherichia coli in these farmers over time, (ii) the genetic relatedness between such E. coli isolates from humans and pigs within the same farm, and (iii) the association between human ESBL carriage and the presence of ESBL-producing E. coli in pigs. To this end, we extended our sampling and subsequent analysis, including ESBL gene typing, plasmid (sub)typing, and E. coli sequence typing, on the same 39 conventional Dutch pig farms on which we have previously studied the association between ESBL carriage in humans and pigs cross-sectionally (4), the role of farm management, including antimicrobial use, on ESBL carriage in pigs (14), and the role of air exposure on within-farm ESBL transmission (15). Overall, the data presented here provide a longitudinal and nationwide overview of the prevalence and molecular characteristics of ESBL-producing E. coli colonizing humans and pigs on Dutch pig farms, suggesting the possibility of clonal and horizontal dissemination of ESBL genes between pigs and pig farmers.

(Results from this study were partially presented in a poster presentation at the 13th International Society for Veterinary Epidemiology and Economics Conference in Maastricht, The Netherlands, 2012 [abstract 12787], in a poster presentation at the 23rd European Congress of Clinical Microbiology and Infectious Diseases in Berlin, Germany, 2013 [P1471], in an oral presentation at the 23rd Conference on Epidemiology in Occupational Health in Utrecht, The Netherlands, 2013 [abstract 328], in a poster presentation at the 53th Interscience Conference on Antimicrobial Agents and Chemotherapy in Denver, CO, USA, 2013 [C2-1610], in an oral presentation at the 3rd International One Health Congress in Amsterdam, The Netherlands, 2015 [abstract 148], in an oral presentation at the 26th European Congress of Clinical Microbiology and Infectious Diseases in Amsterdam, The Netherlands, 2016 [abstract 1944], and in a poster presentation at the 28th annual International Society for Environmental Epidemiology conference in Rome, Italy, 2016 [P3-247].)

## RESULTS

### Prevalence of ESBL-producing E. coli in humans and pigs.

Out of 146 participants, 119 provided samples during all four sampling occasions, 15 during three sampling occasions, and 12 during one or two sampling occasions. ESBL genes were detected in E. coli isolates from 17 participants (13 pig farmers and 4 family members) at one or more sampling occasions (Table 1). From the total of 542 observations in 146 humans, 23 ESBL-positive observations were documented. Human prevalence over all sampling occasions was 4% (confidence interval [CI], 3 to 6%) and ranged from 6% (95% CI, 2 to 9%) at the beginning to 2% (95% CI, 0 to 5%) at the last sampling occasion (8/141, 7/137, 5/135, and 3/129 in time order). Overall, the prevalence of farms where ESBL-producing E. coli was present in pigs was 34% and ranged from 44% to 28% between the first and last sampling occasions (17/39, 13/39, 12/39, and 11/39 in time order). Out of 39 farms, 18 (46%) were positive for ESBL-producing E. coli in pigs at one or more sampling occasions, of which 9 farms (23%) were positive at all four sampling occasions. In 21 farms (54%), pigs were negative for ESBL-producing E. coli isolates at all sampling occasions.

**TABLE 1:** ESBL genes in human and pig isolates in pig farms (only positive observations are listed)

Farm	Origin	ESBL gene^*a*^			
		0 mo^*b*^	6 mo	12 mo	18 mo
1^*c*^	Farmer		*bla*_CTX-M-1_ (2)	*bla*_CTX-M-1_ (1)	*bla*_CTX-M-1_ (3)
	Pigs	*bla*_CTX-M-1_ (16)	*bla*_CTX-M-1_ (12)	*bla*_CTX-M-1_ (3)	*bla*_CTX-M-1_ (10)
2	Pigs	*bla*_CTX-M-1_ (1)			
3	Pigs	*bla*_CTX-M-1_ (1) *bla*_CTX-M-2_ (4) *bla*_TEM-52c var_ (6)	*bla*_CTX-M-2_ (2) *bla*_TEM-52c_ (2) *bla*_TEM-52c var_ (1)	*bla*_CTX-M-1_ (1) *bla*_TEM-52c_ (4)	*bla*_CTX-M-2_ (1) *bla*_TEM-52c_ (8)
4^*c*^	Farmer A	*bla*_CTX-M-1_ (1)			
	Farmer B	*bla*_CTX-M-1_ (1)	*bla*_CTX-M-1_ (1)		
	Pigs	*bla*_CTX-M-1_ (7)	*bla*_CTX-M-1_ (6)	*bla*_CTX-M-1_ (3)	
5^*c*^	Farmer		*bla*_CTX-M-1_ (2)		*bla*_CTX-M-14_ (1)
	Pigs	*bla*_CTX-M-1_ (1) *bla*_CTX-M-14_ (10)	*bla*_CTX-M-1_ (3) *bla*_CTX-M-14_ (2)	*bla*_CTX-M-1_ (1) *bla*_CTX-M-14_ (2)	*bla*_CTX-M-1_ (2) *bla*_CTX-M-14_ (3) *bla*_TEM-52c_ (1)
6	Pigs	*bla*_CTX-M-1_ (4)			
7	Pigs	*bla*_CTX-M-14_ (1)			
8	Pigs	*bla*_CTX-M-1_ (16)	*bla*_CTX-M-1_ (13)	*bla*_CTX-M-1_ (13)	*bla*_CTX-M-1_ (8)
9^*c*^	Farmer	*bla*_CTX-M-1_ (1)	*bla*_CTX-M-15_ (1)		No sample
	Pigs	*bla*_CTX-M-1_ (13)	*bla*_CTX-M-1_ (7) *bla*_CTX-M-15_ (1)	*bla*_CTX-M-1_ (9)	*bla*_CTX-M-1_ (3)
10^*c*^	Farmer			*bla*_CTX-M-1_ (2)	
	Pigs			*bla*_CTX-M-1_ (9)	*bla*_CTX-M-1_ (12)
11	Farmer		*bla*_CTX-M-15_ (1)		
12	Pigs	*bla*_TEM-52c_ (1)	*bla*_CTX-M-1_ (1) *bla*_TEM-52c_ (1)		*bla*_TEM-52c_ (5)
13	Pigs	*bla*_CTX-M-15_ (11)	*bla*_CTX-M-1_ (1)		
			*bla*_CTX-M-15_ (2)		
14	Family member		*bla*_CTX-M-1_ (2)		No sample
15	Farmer				*bla*_CTX-M-2_ (1)
16^*c*^	Farmer		*bla*_CTX-M-1_ (1)		
	Pigs	*bla*_CTX-M-1_ (6)	*bla*_CTX-M-1_ (9)	*bla*_CTX-M-1_ (8)	*bla*_CTX-M-1_ (6)
17	Family member			*bla*_CTX-M-1_ (1)	
18^*c*^	Farmer A			*bla*_TEM-52c var_ (1)	
	Farmer B	*bla*_TEM-52c var_ (2)			
	Pigs	*bla*_CTX-M-1_ (1) *bla*_TEM-52c var_ (14)	*bla*_CTX-M-1_ (1) *bla*_TEM-52c var_ (11)	*bla*_CTX-M-1_ (1) *bla*_TEM-52c var_ (10)	*bla*_TEM-52c_ (3) *bla*_TEM-52c var_ (1)
19^*c*^	Farmer	*bla*_CTX-M-1_ (1)		*bla*_CTX-M-1_ (2)	
	Family member	*bla*_CTX-M-1_ (1)			
	Pigs	*bla*_CTX-M-1_ (10) *bla*_CTX-M-32_ (1)	*bla*_CTX-M-1_ (6)	*bla*_CTX-M-1_ (5)	*bla*_CTX-M-1_ (2)
20	Pigs	*bla*_CTX-M-1_ (11) *bla*_TEM-52c_ (2)	*bla*_CTX-M-1_ (6)	*bla*_CTX-M-1_ (7) *bla*_TEM-52c_ (1)	*bla*_CTX-M-1_ (5) *bla*_TEM-52c_ (2)
21	Pigs	*bla*_TEM-52c_ (1)			
22^*c*^	Farmer	*bla*_CTX-M-14_ (2)			
	Pigs	*bla*_CTX-M-14_ (7)	*bla*_CTX-M-1_ (1) *bla*_CTX-M-14_ (1)	*bla*_CTX-M-14_ (1)	
23	Family member	*bla*_CTX-M-1_ (3)			

a The numbers within parentheses indicate the total number of distinctive colonial morphotypes tested that carried the specific ESBL gene.

b All human and pig *bla*_CTX-M-1_-carrying isolates collected at 0 months were not tested for the presence of additional genes.

c Within this farm the presence of the same ESBL genes was established in both pig farmers and their pigs on one or more sampling occasion(s).

### ESBL gene epidemiology.

In total, 417 ESBL-producing E. coli isolates were recovered on four sampling occasions, of which 34 were from the 17 human participants and 383 were from pigs. Among them, four ESBL gene types were identified belonging to either the *bla*_CTX-M_ or *bla*_TEM_ gene family. The *bla*_CTX-M-1_ gene was predominant among the recovered isolates and was detected in 25 out of 34 human (74%) and 261 out of 383 pig isolates (68%). Twelve out of 17 (71%) participants positive for the presence of ESBL-producing E. coli carried the *bla*_CTX-M-1_ gene at least once. Other frequently found ESBL genes were *bla*_TEM-52c_/*bla*_TEM-52c var_ (*n* = 74 [19%] and *n* = 3 [9%] isolates in pigs and humans, respectively), *bla*_CTX-M-14_ (*n* = 27 [7%] and *n* = 3 [9%]), and *bla*_CTX-M-15_ (*n* = 14 [4%] and *n* = 2 [6%]). On several farms, diverse ESBL gene types were found in pigs on the same sampling occasion and/or the present gene types could differ over time within the same farm. On nine farms, ESBL genes were detected in both isolates from pigs and farmers on the same sampling occasion. On eight of these farms, the ESBL gene type found in pig farmers was identical to the predominantly (*n* = 3) or exclusively (*n* = 5) detected ESBL gene type in pig isolates of the same farm. Detailed data regarding ESBL genes in humans and pigs per farm and sampling occasion are listed in Table 1.

### Plasmid (sub)types and E. coli sequence types.

A total of 104 ESBL-harboring isolates (34 human and 70 pig isolates) were selected for further genetic characterization of plasmids and strains. This selection included isolates detected on nine farms where ESBL genes were present in both humans and pigs. From these farms, all human ESBL-producing E. coli isolates (*n* = 26) and a maximum of five randomly selected identical ESBL gene-harboring E. coli isolates from pigs within the same farm were included (Table 2). In addition, human derived ESBL-producing E. coli isolates detected in five farms without ESBL genes present in their pigs were included (*n* = 8) (Table 3).

**TABLE 2:** Plasmid (sub)types and E. coli sequence types of human and pig isolates within the same farm

Farm	Origin	Sampling occasion											
		0 mo			6 mo			12 mo			18 mo		
		Gene	Plasmid^*a*^	ST (no. of isolates)	Gene	Plasmid	ST (no. of isolates)	Gene	Plasmid	ST (no. of isolates)	Gene	Plasmid	ST (no. of isolates)
1	Human				*bla*_CTX-M-1_	IncI1 (7/7)	48 (1)	*bla*_CTX-M-1_	IncI1 (3/3)	10 (1)	*bla*_CTX-M-1_	IncI1 (3/3)	1486 (1)
					*bla*_CTX-M-1_	IncI1 (7/7)	1670 (1)				*bla*_CTX-M-1_	IncI1 (7/7)	48 (1)
											*bla*_CTX-M-1_	IncI1 (7/7)	1670 (1)
	Pigs				*bla*_CTX-M-1_	IncI1 (3/3)	58 (3)	*bla*_CTX-M-1_	IncI1 (3/3)	58 (1)	*bla*_CTX-M-1_	IncI1 (3/3)	1486 (1)
					*bla*_CTX-M-1_	IncI1 (3/3)	398 (1)	*bla*_CTX-M-1_	IncI1 (3/3)	398 (1)	*bla*_CTX-M-1_	IncI1 (3/3)	1952 (1)
					*bla*_CTX-M-1_	IncI1 (3/3)	410 (1)	*bla*_CTX-M-1_	IncI1 (38/3)	58 (1)	*bla*_CTX-M-1_	IncI1 (7/7)	58 (1)
											*bla*_CTX-M-1_	IncI1 (7/7)	398 (1)
											*bla*_CTX-M-1_	IncI1 (38/3)	1486 (1)
4	Human a	*bla*_CTX-M-1_	IncI1 (3/3)	453 (1)^*b*^	*bla*_CTX-M-1_	IncI1 (3/3)	453 (1)						
	Human b	*bla*_CTX-M-1_	IncI1 (3/3)	453 (1)									
	Pigs	*bla*_CTX-M-1_	IncI1 (3/3)	453 (1)	*bla*_CTX-M-1_	IncI1 (3/3)	453 (1)						
		*bla*_CTX-M-1_	IncI1 (3/3)	453 (2)^*b*^	*bla*_CTX-M-1_	IncI1 (38/3)	453 (4)						
		*bla*_CTX-M-1_	IncI1 (7/7)	58 (1)									
		*bla*_CTX-M-1_	IncI1 (7/7)	453 (1)									
5	Human				*bla*_CTX-M-1_	IncI1 (7/7)	48 (2)				*bla*_CTX-M-14_	F2:A-:B-	10 (1)
	Pigs				*bla*_CTX-M-1_	IncI1 (3/3)	58 (2)				*bla*_CTX-M-14_	F2:A-:B-	10 (1)
					*bla*_CTX-M-1_	IncI1 (278_SLV_/-)^*c*^	1607 (1)				*bla*_CTX-M-14_	F2:A-:B-	2064 (1)
											*bla*_CTX-M-14_	F2:A-:B-	227 (1)
9	Human	*bla*_CTX-M-1_	IncI1 (7/7)	540 (1)	*bla*_CTX-M-15_	F-:A20:B1	12 (1)						
	Pigs	*bla*_CTX-M-1_	IncI1 (7/7)	540 (2)	*bla*_CTX-M-15_	Chromosome	12 (1)						
		*bla*_CTX-M-1_	IncI1 (7/7)	101 (1)									
		*bla*_CTX-M-1_	IncI1 (7/7)	6593 (1)									
		*bla*_CTX-M-1_	IncI1 (7/7)	10_SLV_ (1)									
10	Human							*bla*_CTX-M-1_	IncI1 (7/7)	6593 (1)			
								*bla*_CTX-M-1_	IncI1 (7/7)	6404 (1)			
	Pigs							*bla*_CTX-M-1_	IncI1 (7/7)	6404 (1)			
								*bla*_CTX-M-1_	IncI1 (7/7)	6587 (1)			
								*bla*_CTX-M-1_	IncI1 (7/7)	101 (3)			
16	Human				*bla*_CTX-M-1_	IncI1 (7/7)	910 (1)						
	Pigs				*bla*_CTX-M-1_	IncI1 (7/7)	328 (1)						
					*bla*_CTX-M-1_	IncI1 (7/7)	638 (1)						
					*bla*_CTX-M-1_	IncI1 (7/7)	4040 (1)						
					*bla*_CTX-M-1_	IncI1 (7/7)	6859_SLV_ (1)						
					*bla*_CTX-M-1_	IncI1 (7/7)	7618_SLV_ (1)						
18	Human a	*bla*_TEM-52c var_	X1	10 (2)									
	Human b							*bla*_TEM-52c var_	X1	302 (1)			
	Pigs	*bla*_TEM-52c var_	X1	10 (1)				*bla*_TEM-52c var_	X1	302 (1)			
		*bla*_TEM-52c var_	X1	101 (1)				*bla*_TEM-52c var_	X1	10 (3)			
		*bla*_TEM-52c var_	X1	154 (1)				*bla*_TEM-52c var_	X1	5579 (1)			
		*bla*_TEM-52c var_	X1	877 (1)									
		*bla*_TEM-52c var_	X1	5579 (1)									
19	Human a	*bla*_CTX-M-1_	IncI1 (7/7)	711 (1)^*b*^				*bla*_CTX-M-1_	IncI1 (7/7)	227 (2)			
	Human b	*bla*_CTX-M-1_	IncI1 (7/7)	101 (1)									
	Pigs	*bla*_CTX-M-1_	IncI1 (7/7)	711 (1)				*bla*_CTX-M-1_	IncI1 (7/7)	227 (1)			
		*bla*_CTX-M-1_	IncI1 (7/7)	10 (1)				*bla*_CTX-M-1_	IncI1 (7/7)	88 (1)			
		*bla*_CTX-M-1_	IncI1 (7/7)	227 (2)				*bla*_CTX-M-1_	IncI1 (7/7)	218 (1)			
		*bla*_CTX-M-1_	IncI1 (7/7)	3321 (1)^*b*^				*bla*_CTX-M-1_	IncI1 (7/7)	711 (2)			
22	Human	*bla*_CTX-M-14_	F2:A-:B-	48 (1)									
		*bla*_CTX-M-14_	ColE2	3079 (1)									
	Pigs	*bla*_CTX-M-14_	F2:A-:B-	744 (2)									
		*bla*_CTX-M-14_	F2:A-:B-	3595 (1)									
		*bla*_CTX-M-14_	F2:A-:B13	48 (1)									
		*bla*_CTX-M-14_	F2:A-:B42	2946_SLV_ (1)									

a For plasmids belonging to the IncI1 group, we report the plasmid sequence type (pST) and plasmid clonal complex (pCC) within parentheses following the format pST/pCC.

b Plasmid subtype and MLST were constructed from whole-genome sequencing (20).

c SLV, single locus variant.

**TABLE 3:** Plasmid subtypes and E. coli sequence types of human isolates obtained from farms without ESBL producer-harboring pig isolates

Source	Sampling occasion	Gene	Plasmid^*a*^	ST (no. of isolates)
Human (farm 11)	6 mo	*bla*_CTX-M-15_	Chromosome	131 (1)
Human (farm 14)	6 mo	*bla*_CTX-M-1_	IncI1 (58/58)	34 (1)
		*bla*_CTX-M-1_	IncI1 (58/58)	23 (1)
Human (farm 15)	6 mo	*bla*_CTX-M-2_	IncI1 (12/12)	58 (1)
Human (farm 17)	12 mo	*bla*_CTX-M-1_	IncI1 (3/3)	23_SLV_ (1)
Human (farm 23)	0 mo	*bla*_CTX-M-1_	F2:A-:B-	86 (1)
		*bla*_CTX-M-1_	IncI1 (3/3)	58 (1)
		*bla*_CTX-M-1_	IncI1 nontypeable^*b*^	86 (1)

a For plasmid belonging to IncI1 group we report the plasmid sequence type (pST) and plasmid clonal complex (pCC) within parentheses as (pST/pCC).

b Exact matches found only for 4 out of 5 loci included in the plasmid MLST scheme (*repI1* [2]-*ardA* [1]-*trbA* [4]-*pilL* [2]), while no positive PCR was obtained for the *sogS* gene after repeated attempts. The partial allelic profile obtained could correspond to pST-3/pCC-3, pST-101, or pST-220 based on the IncI1 plasmid MLST database (https://pubmlst.org/bigsdb?db=pubmlst_plasmid_seqdef).

In seven out of the nine farms, at least one of the human and one of the pig isolates on the same farm and sampling occasion were identical in gene, plasmid (sub)type, and E. coli sequence type (ST) (farms 1, 4, 5, 9, 10, 18, and 19). On four farms, human- and pig-derived ESBL-harboring isolates were identical in gene and plasmid subtype on the same sampling occasion (farms 1, 16, 19, and 22).

On farms 1, 4, 5, 9, 10, 16, and 19, *bla*_CTX-M-1_ (*n* = 70) was identified in humans and pigs, always carried on IncI1 plasmid subtypes: pST7/pCC7 (*n* = 43 [61%]), pST3/pCC3 (*n* = 20 [28%]), pST38/pCC3 (*n* = 6 [9%]), and pST278_SLV_ (*n* = 1 [2%]) (where SLV is single locus variant). On farms 10, 16, and 19, plasmid subtype pST7/pCC7 was exclusively identified in human- and pig-derived isolates. On farms 5 and 22, *bla*_CTX-M-14_ (*n* = 11) was detected in humans and pigs located on plasmids assigned to F2:A-:B- (*n* = 8), F2:A-:B13 (*n* = 1), F2:A-:B42 (*n* = 1), and ColE2 (*n* = 1) (sub)types. On farm 18, the *bla*_TEM-52c var_ gene was detected in isolates from both humans and pigs, in all of which the gene was present on IncX1 plasmids (*n* = 13). On farm 9, *bla*_CTX-M-15_ (*n* = 2) was detected in one human- and one pig-derived isolate, located on an F-:A20:B1 plasmid subtype and the chromosome, respectively.

E. coli isolates were distributed into 38 different STs, each comprised of 1 to 12 isolates, with ST453/clonal complex 86 (CC86) (*n* = 12), ST10/CC10 (*n* = 10), and ST58/CC155 (*n* = 9) being the predominant ones, while isolates belonging to human-related epidemic clones (i.e., ST101, ST410, ST711, and ST744) were also identified. The number of identified STs per farm varied between one (farm 9) and eight (farm 1), with the presence of diverse E. coli STs circulating within the majority of the farms. On farm 4, 12 out of the 13 isolates harboring *bla*_CTX-M-1_ on different incI1 plasmid types obtained during two sampling occasions belonged to ST453.

On five farms (farms 11, 14, 15, 17, and 23), human participants carried an ESBL producer without ESBL producers being present in pigs on the farm (Table 3). In these isolates, the ESBL genes were located on diverse IncI1 plasmid subtypes (pST3/pCC3, pST58/pCC58, pST12/pCC12, and a nontypeable pST), an F plasmid subtype (F2:A-:B-), and the chromosome. In these participants, six different STs were detected, namely, ST23, ST23_SLV_, ST34, ST58, ST86, and ST131.

### Prolonged carriage of ESBL-producing E. coli in humans.

Out of 134 participants with at least three analyzed samples, five pig farmers from five different farms carried an ESBL-producing E. coli strain more than once. Three out of these five pig farmers carried the same ESBL gene (*bla*_CTX-M-1_) more than once. For two out of these three pig farmers (farm 1 and farm 4), isolates involved belonged to identical STs and carried *bla*_CTX-M-1_ on identical plasmid subtypes in different sampling occasions (Table 2). For one pig farmer (farm 19), only the *bla*_CTX-M-1_-carrying plasmid subtype was identical over time. For the two remaining farmers, different genes, strains, and plasmid subtypes were observed over time. The genetic characteristics of the ESBL-producing E. coli from humans and their pigs are listed in Table 2.

### Association between ESBL-producing E. coli in humans and pigs.

Out of the total of 146 human participants, 134 provided a fecal sample during at least three sampling occasions. Therefore, 22 out of 542 human observations were excluded from further analysis, which were all negative for ESBL. Characteristics of human participants are listed in Table 4.

**TABLE 4:** Baseline characteristics of participants who obtained at least three samples (*n* = 132)^*a*^

Human characteristic	Frequency (%)
Gender	
Male	77 (58)
Female	55 (42)
Category	
Farmer	45 (34)
Family of farmer	70 (53)
Employee	17 (13)
Age (yrs)	132 (mean, 36; range, 7–79)
<18	30 (23)
18–65	99 (75)
>65	3 (2)
Avg no. of hours working in the farm per wk	126 (mean, 25; range, 0–80)
0	39 (31)
1–20	26 (21)
≥20	61 (48)
Smoking	
Yes	11 (8)
No	121 (92)

a Measured at the start of the study period (first sampling occasion). Differences in characteristics between the first and further sampling occasions were minor. Two out of 134 participants did not provide a sample at the first sampling occasion.

From the total of the 17 human ESBL carriers, 12 (10 pig farmers and 2 of their family members) were living and working on nine farms, where ESBL-positive isolates from pigs were collected as well on the same sampling occasion (18 human ESBL-positive observations). The remaining five human ESBL carriers were living on five farms where ESBL-producing E. coli was not detected in pigs. Human ESBL carriage was univariately associated with the presence of ESBL-producing E. coli in pigs (odds ratio [OR] = 8.9 and 95% CI = 2.9 to 27.4) and the average number of hours working on the pig farm per week (OR = 1.04 and 95% CI = 1.01 to 1.06). Of the confounders considered, age and gender were significantly associated univariately (OR = 1.04 and 95% CI = 1.01 to 1.07 and OR = 5.3 and 95% CI = 1.4 to 19.9, respectively). Both age and gender were moderately correlated with average number of hours working on the pig farm per week (ρ = 0.49 and ρ = 0.54, respectively). After mutual adjustment in bivariate analyses, the association between human ESBL carriage and average number of working hours on the pig farm per week remained, while the association with age and gender became statistically nonsignificant. To avoid overadjustment in multivariate analysis, only average number of working hours on the pig farm per week was considered for multivariate analysis in addition to the presence of ESBL-producing E. coli in pigs. Results of univariate analysis are presented in Table 5.

**TABLE 5:** Longitudinal univariate and multivariate analyses for ESBL carriage in pig farmers, family members and employees

Determinant	No.^*a*^ or mean	Univariate or CI	Multivariate or CI
Presence of ESBL in pigs			
Yes	159	8.9 (2.9–27.4)	8.7 (3.0–25.6)
No	362	Ref.^*b*^	Ref.
Avg no. of hours working on pig farm per wk (per h)	24 ± 24	1.04 (1.01–1.06)	1.04 (1.01–1.06)
Per 10 h		1.4 (1.1–1.7)	1.4 (1.2–1.7)
Age (per yr)	36 ± 17	1.04 (1.01–1.07)	
Per 10 yrs		1.45 (1.05–1.99)	
Gender			
Male	302	5.3 (1.4–19.9)	
Female	219	Ref.	
Smoking			
Yes	37	1.5 (0.2–12.9)	
No	473	Ref.	

a Based on total number of observations.

b Ref., reference category.

In multivariate analysis, human ESBL carriage was associated with both presence of ESBL-producing E. coli in pigs (OR = 8.7 and 95% CI = 3.5 to 25.6) and average number of hours working on the pig farm per week (OR = 1.04 and 95% CI = 1.02 to 1.06). The final model is presented in Table 5. When restricting the final model to isolates harboring predominant ESBL gene *bla*_CTX-M-1_ (16 positive observations from 12 participants), both associations remained (presence of *bla*_CTX-M-1_ in pigs [OR = 14.0 and 95% CI = 3.5 to 55.6] and average number of hours working on the pig farm per week [OR = 1.02 and 95% CI = 1.00 to 1.05]).

## DISCUSSION

The number of farms with ESBL-producing E. coli present in pigs declined over time. Similarly, we observed a downward trend in the prevalence of such isolates among humans living and working on these pig farms, probably because of their reduced exposure via their pigs. Human prevalence ranged from 6% to 2% during the study period, which is roughly comparable to the prevalence in the Dutch general population (13, 16). Since 12 out of the 17 (71%) participants positive for the presence of ESBL-producing E. coli carried the *bla*_CTX-M-1_ gene at least once, *bla*_CTX-M-1_ was the most frequently observed gene in humans. In contrast, a low proportion of carriage of *bla*_CTX-M-1_ gene among the general population in The Netherlands was observed. In previous Dutch studies among 1,695 residents of Amsterdam, 2,432 residents living in the vicinity of livestock farms, and 4,177 residents of The Netherlands, the proportion of *bla*_CTX-M-1_ gene carriage ranged from 12 to 18% (13, 16, 17). In addition, these studies revealed that the human-related *bla*_CTX-M-15_ gene was the most frequent gene among the Dutch general population, whereas in our study, this gene was found in only two participants, underscoring that pig farmers and the general population differ on their ESBL gene type carriage (18).

In most cases, the ESBL gene type found in pig farmers was similar to the predominantly or exclusively detected ESBL gene type in the pig isolates from the same farm (Table 1). For seven farms, E. coli isolates with an identical combination of a mostly non-human-specific ESBL gene, plasmid (sub)type, and strain ST were observed in human and pig isolates from the same sampling occasion. This is suggestive of a potential clonal transmission, especially given that these E. coli isolates with identical molecular characteristics were obtained, within the closely epidemiologically linked cluster of a farm, more frequently than expected by chance. Of note, we have previously shown that E. coli strains exhibiting the combination of identical β-lactamase gene, plasmid (sub)type, and strain ST and being recovered within epidemiologically linked clusters (i.e., Dutch households) belong to identical pulsed-field gel electrophoresis (PFGE) types (19). In addition, we have previously documented the presence of only six single nucleotide polymorphisms (SNPs) among a subset of the E. coli isolates exhibiting identical molecular characteristics and described here (farm 4 for the first sampling occasion) (20). This low number of SNPs among our isolates is comparable to the genetic similarity seen between isolates within an outbreak, confirming a within-farm clonal transmission between farmer and their pigs. Considering that *bla*_CTX-M-1_ is frequently found in livestock (3, 5, 7, 21), transmission from animals to humans is likely to occur. However, the coexistence of *bla*_CTX-M-15_ in both a human and pooled pig sample within one farm suggests that transmission could be bidirectional. Although the data generated in this study taken together with our previous studies are suggestive of within-farm clonal transmission, further high-resolution typing with whole-genome sequencing is needed to confirm that hypothesis.

On four farms, an identical plasmid (sub)type carrying a specific ESBL gene was observed among nonclonally related E. coli strains belonging to diverse STs, highlighting the potential involvement of horizontally transferred plasmids in the within-farm epidemiology. Of note, we have previously confirmed horizontal plasmid transfer among our diverse ST E. coli isolates recovered from farm 19 solely for the first sampling occasion by showing the absence of any SNPs for their ESBL-encoding plasmids (20). However, the confirmation of the within-farm horizontal plasmid transfer warrants whole-genome sequence typing of the recovered E. coli isolates and assessing the potential for conjugal transfer of the ESBL-encoding plasmids.

The presence of ESBL-producing E. coli in humans was associated with the presence of ESBL-producing E. coli in pigs, with both the presence of ESBL-carrying pigs in the farm and the duration of exposure being relevant for human carriage of ESBL-producing E. coli. Considering the daily intensive contact of animal caretakers with livestock, clonal transmission is likely to occur between animals and animal caretakers. This is supported by the epidemiological association found in this study, combined with the genetic similarities of ESBL-producing E. coli isolates obtained in humans and pigs within the same farm. At the same time, a great diversity in plasmid (sub)types and strain STs of ESBL-producing E. coli isolates from both humans and pigs was observed between farms.

Only 2 pig farmers out of the 134 people living and/or working on pig farms carried isolates with identical combinations of ESBL gene, plasmid (sub)type, and strain ST over time. For one of these pig farmers (farm 4), the isolates found in pigs exhibited identical molecular characteristics over time as well. Persistent carriage and repeated transmission events due to ongoing exposure to ESBL producers in pigs can both be possible explanations for these observations. Duration of ESBL producer carriage can be dependent on strain factors. In a Dutch study in the general population, isolates harboring *bla*_CTX-M-1_ were lost more easily than other gene types (12). In a health care setting, ESBL-producing E. coli clone ST131 was more persistent than other ESBL-producing E. coli strains (22). Several of the STs of E. coli from human origin detected in our study within farms where ESBL-producing isolates were present in pigs (ST10, ST48, ST227, ST302, ST453, ST540, ST711, ST1486, and ST1670) have been previously associated with E. coli from porcine origin, whereas they have only incidentally been described for E. coli from humans (23–28). Within-pig farm epidemiology of ESBL-producing E. coli is mostly facilitated by animal-related STs with potentially low capability of colonization and maintenance in human enteric cavity, leading to low prevalence and a low percentage of persistence among pig farmers in spite of their close contact with pigs.

To the best of our knowledge, this is the first longitudinal study on a large number of farms providing the molecular characteristics of ESBL-producing E. coli in both pigs and pig farmers repeatedly over time. Great diversity was seen at the level of gene, plasmid, and strain in human- and pig-derived isolates within and between farms and over time, which highlights the complex and dynamic epidemiology of ESBL-producing E. coli within Dutch pig farms. However, the within-farm genetic similarity in ESBL genes, plasmid (sub)types, and/or E. coli STs could suggest the possibility of clonal and horizontal dissemination of ESBL genes between pigs and pig farmers, considering the intensive daily contact between animals and their caretakers. Our longitudinal data highlight that prolonged carriage of ESBL-producing E. coli in pig farmers was observed only incidentally.

## MATERIALS AND METHODS

### Study design and fecal sample collection.

A total of 39 conventional pig farms located in The Netherlands were enrolled between 2011 and 2013. During four repeated sampling occasions with a 6-month period, fecal samples were obtained from 146 farmers, their family members, and employees on a voluntary basis from 33 out of these 39 farms. Human fecal samples were self-collected using sterile plastic containers and sent to the laboratory accompanied by self-reported questionnaires on general characteristics, farm activities, and duration of animal contact. In addition, from all 39 farms at each sampling occasion, rectal swabs from 60 pigs were collected by veterinarians using sterile cotton-wool swabs and sent refrigerated to the laboratory by courier. All animal categories (sows, gilts, suckling piglets, weaning piglets, and finishing pigs) present on the farm were sampled. Rectal swabs were pooled into 10 pools (2 pools per age category) of six pigs per farm. When no finishing pigs were present, weaning piglets were sampled extra instead. Each pooled sample consisted of 6 samples taken from a single animal category out of the same pen. Given the time interval of 6 months between the sampling occasions and the average life span in the farm, it is expected that the majority of the animals present were sampled during only one occasion.

### Laboratory analysis.

Both human and pooled pig samples were screened for the presence of ESBL-producing E. coli by enrichment in 10 mL buffered peptone water followed by plating on selective agar medium (Brilliance ESBL agar; Oxoid, Basingstoke, UK) and incubation at 37°C aerobically. Bacterial species identification was confirmed using matrix-assisted laser desorption ionization–time of flight mass spectrometry (MALDI-TOF MS; Brucker, Coventry, United Kingdom) according to the manufacturer’s recommendations. Presumptive ESBL-producing distinctive E. coli colonial morphotypes (one to four per sample) were screened for ESBL genes by microarray analysis (Check-MDR CT-101; Check-points, Wageningen, The Netherlands), PCR, and subsequent Sanger sequencing as previously described (4, 29). All recovered ESBL-harboring isolates from humans and a maximum of five (similar ESBL gene-harboring) isolates from their pigs were selected for further plasmid and clonal analysis to, respectively, assess the genetic similarities on the plasmid and strain levels. Plasmids carrying the ESBL genes were identified using a transformation-based approach (29) and the PBRT kit PCR-based replicon typing (Diatheva, Fano, Italy) according to the manufacturer’s recommendations. When necessary, chromosomal location of the ESBL genes was confirmed by I-Ceu-I PFGE and subsequent Southern blot hybridization using intragenic beta-lactamase and 16S rRNA gene probes, as previously described (29). Plasmid multilocus sequence typing (pMLST) and replicon sequence typing (RST) were used, respectively, to further characterize plasmids assigned to I1 and F replicon types as previously described (30, 31). E. coli isolates were characterized by multilocus sequence typing (MLST) based on the combination of alleles for their seven housekeeping genes using the EnteroBase database (https://enterobase.warwick.ac.uk/species/ecoli/allele_st_search) (32).

### Statistical analysis.

Statistical analyses were performed using SAS version 9.4 (SAS Institute, Inc., Cary, NC, USA). Descriptive analyses were used to explore presence of ESBL genes in E. coli isolates from humans and pigs over time. Farms were classified as ESBL producer positive when ESBL was determined in at least one obtained pig isolate. Generalized linear mixed models (PROC GLIMMIX; SAS Institute, Inc.) adjusted for clustering at the farm level and repeated measurements were used to calculate associations between ESBL carriage in humans and potential determinants. Only observations from humans that participated during at least three sampling occasions were included in the model. The considered determinants were the presence of ESBL-producing *Enterobacterales* in pigs and the average number of hours working per week, which were analyzed separately as well as together in a model. The potential confounders age, gender, and smoking were analyzed univariately and selected for multivariate analysis when the *P* value was below 0.2. Model assumptions were checked using diagnostic plots.

### Ethics statement.

The Medical Ethical Committee of the University Medical Centre Utrecht reviewed and approved the study protocol under registration number 10-471/K. All participating human individuals gave written informed consent. All animal fecal samples were collected in compliance with the Dutch law for animal welfare and did not fall under the Dutch Experiments on Animals Act (1996) or Directive 2010/63/EU.

### Data availability.

All data needed to evaluate the conclusions of this study are present in the paper and are also available upon request by the authors.
